# Dataset of British English speech recordings for psychoacoustics and speech processing research: The clarity speech corpus

**DOI:** 10.1016/j.dib.2022.107951

**Published:** 2022-02-15

**Authors:** Simone Graetzer, Michael A. Akeroyd, Jon Barker, Trevor J. Cox, John F. Culling, Graham Naylor, Eszter Porter, Rhoddy Viveros-Muñoz

**Affiliations:** a Acoustics Research Centre, University of Salford, United Kingdom; b Hearing Sciences, Mental Health and Clinical Neurosciences, School of Medicine, University of Nottingham, United Kingdom; c Department of Computer Science, University of Sheffield, United Kingdom; d School of Psychology, Cardiff University, United Kingdom; e Hearing Sciences - Scottish Section, Mental Health and Clinical Neurosciences, School of Medicine, University of Nottingham, United Kingdom

**Keywords:** Speech, British English, Audio, Recording, Sentence, Clarity, Machine learning, Intelligibility, Hearing

## Abstract

This paper presents the Clarity Speech Corpus, a publicly available, forty speaker British English speech dataset. The corpus was created for the purpose of running listening tests to gauge speech intelligibility and quality in the Clarity Project, which has the goal of advancing speech signal processing by hearing aids through a series of challenges. The dataset is suitable for machine learning and other uses in speech and hearing technology, acoustics and psychoacoustics. The data comprises recordings of approximately 10,000 sentences drawn from the British National Corpus (BNC) with suitable length, words and grammatical construction for speech intelligibility testing. The collection process involved the selection of a subset of BNC sentences, the recording of these produced by 40 British English speakers, and the processing of these recordings to create individual sentence recordings with associated transcripts and metadata.

## Specifications Table

**Table:** 

Subject	Psychology: Experimental and Cognitive Psychology
Specific subject area	Psychoacoustic testing of speech intelligibility and quality, where the sound has been altered by signal processing and room acoustics.
Type of data	Digital audio files Metadata in *.json format
How data were acquired	The sentences to be spoken were chosen from the British National Corpus [1]. A combination of automated and manual filtering was used to select sentences suitable for speech intelligibility testing. Talkers were screened before recordings were made; the brief was to recruit British English speakers who had accents that were “not strong”. All talkers were voice actors employed by a radio production company based in the North-West of England. The audio signals were recorded as uncompressed PCM files at 44.1 or 48 kHz. In Pro Tools, files were downsampled to 44.1 kHz if necessary and trimmed. Due to COVID-19, recordings were made in domestic environments, supervized online by a qualified audio engineer, so recording conditions varied between the talkers. However, all talkers used equipment appropriate for professional recording and they were in environments with minimal background noise and low reverberation. The audio engineer ensured that there were no strong effects of room acoustics or other issues such as the talker being distant or off-axis from the microphone. The audio engineer also requested that the actor repeat sentences that had been misspoken.
Data format	Raw audio files in wav format Metadata in JavaScript Object Notation (JSON) format
Parameters for data collection	The controled parameters were the gender, English variety and accent of the talkers and the sentences produced.
Description of data collection	An automated method selected sentences from the British National Corpus, followed by a manual process to ensure that these sentences were suitable for speech intelligibility testing. Each of the 40 talkers was asked to record a unique set of just over 250 British National Corpus sentences. They made recordings of themselves producing the sentences while an audio engineer monitored the recordings for quality. The recordings were segmented by the authors into individual sentence audio files and aligned with sentence transcripts. Metadata were generated.
Data source location	Institution: University of Salford City/Town/Region: Salford Country: UK
Data accessibility	Repository name: Figshare Data identification number: 10.17866/rd.salford.16918180 Instructions for accessing these data: Freely available on a Creative Commons Attribution-ShareAlike 4.0 International License but with some restrictions.
Related research article	S. Graetzer, J. Barker, T. J. Cox, M. Akeroyd, J. F. Culling, G. Naylor, E. Porter, R. Viveros Muñoz, Clarity-2021 challenges: Machine learning challenges for advancing hearing aid processing, in Proceedings of the Annual Conference of the International Speech Communication Association, INTERSPEECH 2021, Brno, Czech Republic, 2021. doi: 10.21437/Interspeech.2021-1574

## Value of the Data

• A fundamental experimental method in auditory research is to measure the intelligibility and quality of processed speech that is presented alongside background noise. The effectiveness of such listening experiments is dependent on what speech material is used. This corpus uses more up-to-date and naturalistic English than other large corpora, such as the IEEE Harvard sentences [2]. It also features forty talkers. When used in auditory tests, this will improve the ecological validity and generalisability of the results.
• The sentences were selected to be appropriate for speech intelligibility testing where listeners type or say out loud what they heard. For this reason, the sentence filtering process was designed to exclude sentences that would be problematic in such experiments, e.g., ones that were too short, too long, used uncommon words or were ungrammatical or unusually grammatically complex for spoken language.
• Researchers in signal processing, architectural acoustics, hearing aid development or audio system design could use the sentences to gauge the effect that synthesis, reproduction or processing has on speech intelligibility and quality. In addition, the database may be used by researchers who need high-quality audio recordings of sentences produced by a large number of talkers for other acoustic or psychoacoustic experiments.
• Speech intelligibility and quality testing are needed when developing new approaches to speech transmission, processing or reproduction. Therefore, the data can be used to gain new insights into areas such as speech synthesis, hearing devices, machine learning and speech processing. The data were designed for use with machine learning; hence, the database comprises ten thousand sentences produced by forty talkers.
• An algorithmic approach was taken to select a subset of sentences from the British National Corpus. This subset allows researchers working in speech technology to create and exploit language models for the sentences, to enhance the processing of speech through machine learning.

## 1 Data Description

Each of the approximately 10,000 monaural signals in the database is a recording of one sentence produced by one speaker. Each sentence was selected from the British National Corpus. Forty voice actors read just over 250 sentences each. The audio recordings are available in tar.gz format for download. Included in the tar file are: a manifest md5 checksum text file for checking the data integrity; metadata in the form of a JSON file (Table 1); a readme text file, and licence information. The audio files are available in 32-bit floating point WAV audio format, with a 44.1 kHz sampling rate.

**Table 1:** Description of JSON metadata.

Field name	Description	Example
prompt	Original prompt provided to speaker	"At the moment I never feel I'm working hard enough."
		
prompt_id	BNC_ID comprising a 3 letter code followed by a 5 digit number	"G21_00436″
		
speaker	T followed by a 3 digit No. uniquely identifying the talker	"T037"
		
wavfile	*T*<talker No.>_<BNC ID> wav filename	"T037_G21_00436″
		
index	No.	"10″
		
dot	Detailed orthographic transcription	"At the moment I never feel I\\'m working hard enough"

The speech database is licensed under terms consistent with the Creative Commons Corporation (“Creative Commons”) Attribution-ShareAlike 4.0 International License, with the additional exception that excludes permission to publicly broadcast the voice recordings or processed versions except as part of a research project. A small number of audio examples may be used to illustrate the performance of speech processing as part of disseminating findings of the research.

As Table 1 shows, the metadata provided include: the prompt given to the speaker; prompt ID; speaker information; wav filename; index, and dot transcription for each signal. The dot transcription excludes commas, full stops, quotation marks, hyphens, exclamation and question marks, colons and semicolons, and adds two backslashes to precede apostrophes marking possession or contraction and to full points (‘.’) marking abbreviation, such as ‘Mr.’ or ‘Mrs.’.

## 2 Experimental Design, Materials and Methods

### 2.1 Stage A: sentence selection

The sentences are a subset of the British National Corpus (BNC) XML Edition [1]. The BNC comprises 4049 spoken and written texts, sampled from a wide range of different sources. The BNC is intended to represent “a wide cross-section of British English from the later part of the 20th century, both spoken and written.” The majority of the items are from written works rather than spoken texts, and most were published between 1985 and 1993.

We ran a two-step process to select the items we used.

Step A1: Automated sentence filtering

First, nearly 12,000 sentences were randomly selected from the whole BNC database, excluding any that:

1. Contained fewer than 7 or more than 10 words.
2. Used one or more unusual words, as measured by a word not being in the Kucera and Francis database [3].
3. Used words that might cause offence or upset, as specified in bad_words.txt, bad_words_short.txt [4], e.g., ‘asshole’, ‘molestation’, ‘suck’.
4. Used punctuation that indicated potentially complex grammatical constructions or typographical errors, i.e., punctuation not in this set: '.\",?!:;‘’.
5. Were tagged in the BNC metadata as any of the following: poetry ‘l’ (for verse line); quotations from some other work than the text itself ‘quote’; titles or headings ‘head’, lists ‘list’; bibliographic citations or references ‘bibl’, or captions ‘caption’.
6. Were duplicates.

The aim was to get about 10,000 sentences after the manual filtering in stage A2, which meant that 11,706 sentences were required after stage A1.

Step A2: Manual sentence filtering

The sentences from the automated process were evaluated by two native English-speaking researchers. Sentences were excluded on the basis of the criteria listed below in Table 2. A total of 10.3% of the sentences were excluded, leaving 10,505 sentences suitable for recording.

**Table 2:** Exclusion criteria for manual filtering of sentences.

Code	Definition	Example
R1	Sentence contains an unusual name or place name	AMB_00998: Mould let out an almighty scream and flew into the air.
R2	Sentence fragments (when not explained by common conversational ellipsis and when not quoted)	HA5_00159: ‘Also Swedish, Norwegian, Russian, German and Arabic.’
R3	Hard to understand or misleading in isolation	GVL_00073: He was a girl, with a lovely oval face.
R4	References to potentially traumatic or unpleasant events like death, mourning and abandonment	HNJ_00147: Such a waste of a young life.
R5	Sexual content, e.g., references to prostitution	JXT_02151: She felt her body sink against him.
R6	Archaic or unusual (rare or dialectal) word/phrase/grammar, excessively poetic or technical language, grammatical/spelling errors, uncommon abbreviations, or interjections or question tags	C85_01510: I always thought thee a fly un.’
R7	Sentence preceded by name of character speaking, a word defined, or a question and answer pair	H8M_02105: Me: I usually do not remember touching the thing.

After filtering, the remaining sentences were allocated to batches randomly and written to 40 text files. These were randomly allocated to the forty speakers. Speakers were given 255 sentences to read, allowing some flexibility in case any sentences had to be rejected post-recording, leaving 10,200 sentences.

### 2.2 Stage B: sentence recording

For the actors recording the sentences, there were no restrictions on British English dialect or the actor's place of birth, location of schooling, or age. Gender balance was achieved. The production company making the recordings used existing contacts to recruit the speakers. The actors were all accustomed to making high-quality audio recordings remotely during lockdown. They recorded their speech using professional recording equipment, with a sound engineer monitoring the recordings. Each speaker was supplied with a set of randomly ordered text prompts. The actors were asked to deliver the sentences in the style of a BBC Radio 4 continuity announcer, i.e. in a relatively natural manner, but slightly slower than one might normally speak. The sentences were read one after the other with a short pause in between. When the actor or the monitoring sound engineer noticed misspeaking, the sentence was re-read.

### 2.3 Stage C: processing recordings

The authors segmented the recordings into individual utterances with aligned transcripts. Transcriptions are provided at the sentence level without annotation of internal word or phoneme boundaries. To do the sentence segmentation and alignment, the steps were as follows:

• Step C1: Do semi-automated segmentation and perform alignment with text prompts.
• Step C2: Complete manual segmentation checks.
• Step C3: Equalise the speech level of the recordings.

These are outlined in more detail below. With the exception of manual sentence filtering, these steps are carried out using code developed by the authors and with the aid of Google Speech-to-Text API.

Step C1: Automated segmentation and alignment with text prompts

This was performed using Google Speech-to-Text API. The process was as follows:

• Unsegmented recordings were split into segments using the Google WebRTC voice activity detector [5].
• Segments were sent to google storage bucket (cloud storage).
• Google speech-to-text was run on each segment to generate transcripts.
• Segments were aligned to the list of transcripts on the basis of the Word Error Rate (WER).

The alignment process was designed to accommodate for the fact that speech segments can contain: (i) fragments of a complete utterance (in cases where voice actors inserted pauses); (ii) outtakes, i.e., where the voice actors have made reading errors, or repetitions to improve the intonation, or (iii) extraneous speech, including communication between the actor and sound engineer.

Step C2: Manual segmentation checks

Two of the authors evaluated a subset of signals generated by the automated segmentation method, where the Word Error Rate (WER) indicated that errors may have been made in the segmentation. Error codes E1 to E6 as defined below in Table 3 were used. Patches were made for the automated processing to correct for the errors identified at this stage. At the end of this process, there were 10,188 sentences remaining.

**Table 3:** Codes for manual segmentation checks.

Code	Description	Example
E1	Mis-segmentation: start missing	H85_03085: he said, ‘Yes, that would be it.’ (missing ‘After a bit, …’).
E2	Mis-segmentation: end missing	CJA_02001: The light went out (missing ‘and came back on’)
E3	Mis-segmentation: extra words at start or end, e.g. two sentences or two versions of sentence concatenated	EFU_00434: They had to be soft and woolly. They had
E4	Error in original transcript, which is reproduced with a correction	A03_00290: Can anyone can offer a holiday cottage? (produced as ‘Can anyone offer a holiday cottage?’)
E5	Error in production and no correct outtake; this is repaired by fixing transcript	J1G_00820: No one came on as sub, did they? (produced as ‘No one came on as a sub, did they?’)
E6	Other, e.g. mispronunciation	HWH_01669: This is the so-called accretion concept of income. (accretion mispronounced)

Step C3: Signal equalization

Signals were equalized in Active Speech Level (ASL) mean square energy, using ITU P.56 method B [6,7]. Sound files were written as floating point WAV files.

## Ethics Statement

Informed consent and rights to release the voice recordings was obtained from each speaker. The actors were paid for their work. Ethical approval by the local ethics committee was not required for the work.

## CRediT authorship contribution statement

**Simone Graetzer:** Conceptualization, Methodology, Data curation, Investigation, Writing – original draft, Project administration. **Michael A. Akeroyd:** Conceptualization, Methodology, Supervision, Funding acquisition, Writing – review & editing. **Jon Barker:** Conceptualization, Methodology, Software, Resources, Validation, Supervision, Funding acquisition, Data curation, Writing – original draft, Writing – review & editing. **Trevor J. Cox:** Conceptualization, Methodology, Resources, Supervision, Funding acquisition, Writing – original draft, Writing – review & editing. **John F. Culling:** Conceptualization, Methodology, Supervision, Funding acquisition, Writing – review & editing. **Graham Naylor:** Conceptualization, Methodology, Supervision, Funding acquisition, Writing – review & editing. **Eszter Porter:** Methodology. **Rhoddy Viveros-Muñoz:** Methodology.

## Declaration of Competing Interest

The authors declare that they have no known competing financial interests or personal relationships which have or could be perceived to have influenced the work reported in this article.
